# NOVEL PLASMA PROTEIN BIOMARKERS: A TIME-DEPENDENT PREDICTIVE MODEL FOR ALZHEIMER’S DISEASE

**DOI:** 10.1093/geroni/igae098.3678

**Published:** 2024-12-31

**Authors:** Tianchi Zhuang, Ting Xu, Xichenhui Qiu, Yingqi Yang, Xiang Qi, Qin Xu, Minghui Ji

**Affiliations:** nanjing medical university, nanjing, Jiangsu, China (People’s Republic); nanjing medical university, harrison, New Jersey, United States; Shenzhen University, nanjing, Jiangsu, China (People’s Republic); nanjing medical university, nanjing, Jiangsu, China (People’s Republic); New York University, New York, New York, United States; nanjing medical university, nanjing, Jiangsu, China (People’s Republic); nanjing medical university, nanjing, Jiangsu, China (People’s Republic)

## Abstract

The prediction of Alzheimer’s disease (AD) onset is crucial for the efficient disease and the management of its progression. While current diagnostic methods often rely on neuroimaging and cerebrospinal fluid (CSF) biomarkers, there is a growing need for less invasive and more accessible predictive tools. The study aimed to construct a risk predictive model for AD onset using novel plasma protein biomarkers. We analyzed data from 440 participants aged 60 and above from the Alzheimer’s Disease Neuroimaging Initiative cohort. Using a combination of Cox regression, LASSO regression, and cross-validation, we identified seven protein signatures (APOE, CGA, CRP, CCL26, CCL20, NRCAM, PYY) predictive of AD risk. Cox model demonstrated robust predictive performance in AD onset, with area under the curve (AUC) values of 0.77, 0.76, and 0.77 at 4, 6, and 8 years, respectively, and stable up to 12 years (AUC≥0.75). The model’s effectiveness was further validated using time-dependent ROC curves and Kaplan-Meier curves, which showed that low-risk individuals had significantly delayed AD onset compared to the high-risk group (P<.001). All identified protein signatures were significantly associated with AD risk (P<.001) and time of onset (P<.01). However, we observed weak correlations between plasma and CSF protein levels, suggesting potential differences in peripheral and central nervous system biomarker profiles. Additionally, three proteins (APOE, CGA, CRP) showed lower expression in APOE ε4 carriers (P<.05). These findings highlight the complex interplay between genetics and protein expression in AD pathogenesis and concludes that plasma protein signatures have potential as valuable predictors of AD risk.

